# Slower Progression Rates in Lower Limb-Onset ALS

**DOI:** 10.3390/jcm15083096

**Published:** 2026-04-18

**Authors:** Yehuda Shovman, Yossef Lerner, Marc Gotkine

**Affiliations:** 1 Department of Neurology, Hadassah Medical Organization and Faculty of Medicine, Hebrew University of Jerusalem, Jerusalem 91120, Israel; 2 The Concern Foundation Laboratories at the Lautenberg Center for Immunology and Cancer Research, Israel-Canada Medical Research Institute, Faculty of Medicine, Hebrew University of Jerusalem, Jerusalem 91120, Israel

**Keywords:** ALS, ALSFRS-R, mixed-effects models

## Abstract

**Objectives:** The aim of this study was to assess the differences in diagnostic delay and disease progression in people with ALS (PALS) based on site of onset. **Methods:** A retrospective analysis of prospectively collected data was performed, including all PALS seen in the ALS clinic in the Hadassah Medical Center between January 2009 and March 2022. PALS were divided to three groups based on site of onset (upper limb onset—ULO, lower limb onset—LLO, or bulbar onset—BO). A linear mixed-effects model was constructed with the following variables: diagnostic delay, site of onset, age of onset and time since the initial visit. The model was applied to the ALSFRS-R total score and the bulbar and motor subscales. **Results:** Data from 1255 visits of 281 PALS were included in the study. PALS with LLO had longer diagnostic delays than PALS in the BO group. Slower decline of total ALSFRS-R score was observed in younger PALS, and in PALS with LLO when compared with PALS with BO or ULO. The slower decline of ALSFRS-R in PALS with LLO was due to a slower decline in the motor subscale. Longer diagnostic delays were associated with lower total ALSFRS-R scores at the initial visit and with slower rates of decline. **Conclusions:** Comparison among PALS with ULO, LLO and BO revealed differences in the diagnostic delay and in the rate of functional decline, suggesting that differentiating between ULO and LLO ALS may be useful in the stratification of PALS in clinical trials.

## 1. Introduction

Amyotrophic lateral sclerosis (ALS) is a progressive neurodegenerative disease with a median survival of 3–5 years from disease onset [1,2]. ALS affects both upper and lower motor neurons, and may be classified broadly as bulbar-onset (BO) disease presenting with bulbar symptoms such as dysarthria or dysphagia (~25% of cases), and spinal-onset (SO) disease presenting with limb muscle weakness (~70% of cases) [3].

The clinical course in people with ALS (PALS) is heterogeneous, complicating clinical trials that aim to identify effective disease-modifying drugs [1,3]. For example, bulbar-onset is associated with a faster rate of functional decline, and therefore, many studies stratify patients based on the site of onset [1,4,5,6]. Thus, understanding the phenotypic heterogeneity could lead to more effective clinical trials and help guide treating physicians.

A widely accepted measure of ALS-related disability is the revised amyotrophic lateral sclerosis functional rating scale (ALSFRS-R) [7], and the rate of functional decline may be expressed as an ALSFRS-R slope [8,9,10,11,12]. For example, steeper slopes are associated with older age at onset, bulbar onset and shorter time from disease onset [10]. Furthermore, several studies analyzed the rate of decline of the ALSFRS-R subscales [8,10], showing that the analysis of these subscales may offer improved prognostication [8].

Site of onset has been shown to be an important variable in models of ALS progression [8,10,11,12], and PALS are typically divided into BO and SO groups. However, SO PALS may not constitute a uniform group, and an alternative approach is to further subdivide this group based on upper limb onset (ULO) or lower limb onset (LLO) [13]. This distinction may be important in light of the predominantly contiguous spread of ALS and possible differences in the spreading patterns among these groups [14].

We hypothesized that predictive analysis based on ALS progression would be more accurate if the SO category was split into ULO and LLO subtypes. Thus, we investigated differences among PALS with BO, ULO, and LLO. Furthermore, a linear mixed model was constructed to quantify the effects of site of onset and other variables on disease progression, as measured by the ALSFRS-R score and its individual subscales.

## 2. Methods

### 2.1. Study Population

This retrospective study analyzed prospectively collected data from all PALS who visited the ALS clinic in the Hadassah Medical Center between June 2009 and March 2022. The study included demographic data (age at onset, sex), clinical data (time of onset, site of onset, physical examination) and electrophysiologic data that were collected during the first visit, as well as ALSFRS-R scores that were recorded during follow-up visits. We excluded PALS with missing medical records or fewer than 2 visits, and PALS with other sites of onset (such as respiratory onset) or multiple sites of onset. PALS that had two or more affected sites within two weeks of symptom onset were classified as having multiple sites of onset. PALS were grouped according to site of onset (BO, ULO, or LLO), and the site of onset was defined based on patient history that was collected during the initial visit, previous medical documentation, physical examination and electrophysiological findings. The involvement of at least one arm or one leg was sufficient for the definition of ULO or LLO.

Upper motor neuron (UMN) involvement was defined as the clinical presence of UMN signs in the site of onset during the initial visit (increased tendon reflexes or upgoing plantar reflex). Lower motor neuron (LMN) involvement was defined as either the presence of LMN signs in the site of onset during the initial visit (fasciculations or atrophy), or electrophysiological findings corresponding to LMN involvement.

The date of symptom onset was self-reported. The diagnosis date was established based on previous medical documentation. For 50 PALS, the diagnosis date was missing. To infer the diagnosis date, we calculated the difference between the initial visit date and diagnosis date for all other participants (PALS were divided into groups based on site of onset, and the calculation was performed for each group separately) and applied the resulting correction factor to those 50 PALS.

ALSFRS-R scores that were documented up to 5 years from disease onset were included.

### 2.2. Statistical Analysis of Characteristics During the First Visit

To evaluate continuous variables, we performed one-way ANOVA for each variable with subsequent post hoc tests based on Tukey’s HSD. For discrete variables, Fisher’s exact tests were performed across the 3 groups. *p*-values were adjusted for multiple comparisons by using the Benjamini–Hochberg correction. *p*-values lower than 0.05 were considered significant.

### 2.3. Linear Mixed-Effects Model

A linear mixed-effects model was constructed to evaluate the ALSFRS-R slope. We have chosen to evaluate the following covariates: site of onset (which is the focus of the current study); the delay from symptom onset to diagnosis, i.e., diagnostic delay (DD), which was previously shown to significantly affect the ALSFRS-R slope in a similar model [10]; involvement of UMN or LMN at the site of onset at presentation; time since initial visit (measured in months); age at onset (measured in years); and sex.

Six different linear models evaluating different combinations of those covariates were constructed. Comparison of the Akaike information criterion (AIC) values (Supplementary Table S1) revealed that the model that best described the data included the following variables: DD, time since initial visit, onset site, and age at onset.

The final model was constructed as follows: ALSFRS-R ~ Onset site * Months + Age * Months + Diagnostic delay * Months + (1 + Months|Patient). The marginal R^2^ was 0.429, indicating that the fixed effects explained 42.9% of the variance, while the conditional R^2^ was 0.965, indicating that the full model (including patient-specific random effects) explained 96.5% of the variance. Predictive error was modest (RMSE = 2.27; MAE = 1.66).

Following the analysis of the total ALSFRS-R slope, a similar model was used to assess the motor and bulbar subscales of the ALSFRS-R. The motor ALSFRS-R subscale was defined as the sum of scores for the following items: handwriting, cutting food and handling utensils, dressing and hygiene, turning in bed and adjusting bed clothes, walking and climbing stairs. The bulbar ALSFRS-R subscale was defined as the sum of scores for the following items: speech, salivation and swallowing.

DD and time since initial visit were measured in months, and age at onset was measured in years. DD and age were centered to improve the interpretability of the model. *p*-values for individual models were calculated by using the Satterthwaite approximation.

### 2.4. Software

Data processing and analysis were performed in R (v. 4.0.2). ANOVA tests were performed by using the aov function. Linear models were built with the lmerTest (v. 3.1-3) package.

## 3. Results

### 3.1. Study Population

A total of 765 PALS were included in the database. In 689 PALS, the disease manifested at a single site (either BO, ULO or LLO) and at a known timepoint. There were 290 PALS with at least two consecutive visits during the 60-month follow-up period from the disease onset. Outlier analysis revealed nine PALS that had DDs of >36 months, and they were excluded. For the remaining 281 PALS, a total of 1255 recorded visits were included in the downstream analysis. The median follow-up period of the recorded visits included in this study was 11.6 months, with an interquartile range (IQR) of 4.9–14.9 months.

**Figure 1:**
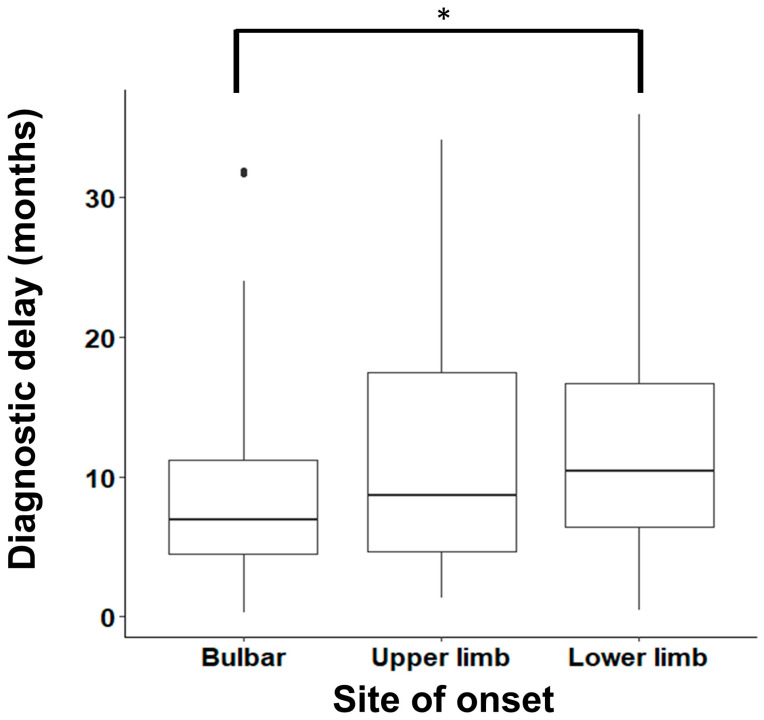
Diagnostic delay in people with ALS, divided by site of onset. A box plot depicting the median diagnostic delay (defined as the difference between the date of symptom onset and the date of diagnosis) in patients with ALS (PALS). PALS were divided by the site of onset. The difference between the lower limb-onset group and the bulbar-onset group is statistically significant (Dunn’s test). Boxes represent IQR; whiskers represent data points up to 2 times IQR. * *p* < 0.005.

A summary of the baseline characteristics at the initial visit is presented in Table 1. There were 100 (35.6%) PALS with ULO, 108 (38.4%) PALS with LLO and 73 (26.0%) PALS with BO. Males constituted 64.1% of all the study population, and the male-to-female ratio was 1.78:1. The average age at onset for all PALS was 57.7 ± 12.1 years, and it was significantly higher (*p* = 0.002) in the BO group (62.2 years) in comparison with the other two groups (55.7 and 56.4 years). The average DD was 10.8 ± 7.9 months, and post hoc analysis showed that it was significantly increased in the LLO group (12.0 ± 8.4 months) in comparison with the BO group (8.7 ± 6.4, *p* = 0.014, Table 2 and Figure 1). In the study population, 212 (77.7%) PALS had both UMN and LMN signs in the site of onset during the initial visit, 49 (17.9%) PALS had only LMN signs, 12 PALS (4.4%) had only UMN signs and data for 8 PALS was missing. The percentage of PALS with mixed UMN and LMN presentation was highest in the BO group (90.3%) compared to the ULO and LLO groups (67.0% and 78.8% respectively; *p* = 0.001).

A total of 236 PALS (84.0%) received treatment with Riluzole, without significant differences across the different groups (*p* = 0.235). Additionally, all PALS were referred to neuro-rehabilitation, in accordance with disease progression.

The average total ALSFRS-R score at the initial visit was 36.3 ± 7.4, and no difference was found across the different groups. As expected, analysis of ALSFRS-R subscales revealed that motor ALSFRS-R scores at the initial visit were significantly decreased in PALS with upper limb onset (ULO) and lower limb onset (LLO) (*p* < 0.001) compared to PALS with bulbar onset (BO). Likewise, initial bulbar ALSFRS-R scores were significantly decreased in the BO group (*p* < 0.001). Initial respiratory ALSFRS-R scores tended to be higher in the ULO group compared to the BO group (*p* = 0.026, Table 2).

### 3.2. Results of Linear Mixed-Effects Model (Total ALSFRS-R Score)

The results of the linear mixed-effects model with the total ALSFRS-R score as the dependent variable are shown in Table 3. The unadjusted average rate of the total ALSFRS-R decline was 1.16 points/month.

The only variable significantly affecting the initial total ALSFRS-R score was DD (*p* = 0.002). The total ALSFRS-R slope was significantly affected by DD, the age at onset and the site of onset. Each additional month of DD decreased the slope by 0.020 points/month (*p* < 0.001), signifying a slower progression. Furthermore, older age was associated with higher slopes, with each additional year at onset increasing the slope by 0.099 points/month (*p* = 0.005).

Regarding the site of onset, no difference in slope was found between the BO and ULO groups (*p* = 0.701). However, a comparison between PALS with BO and PALS with LLO showed that PALS with LLO had a slower progression (0.244 points/month, *p* = 0.027).

When the model was re-run with only ULO and LLO patients, it was found that total ALSFRS-R declined more slowly in LLO patients (a difference of 0.204 points/month, *p* = 0.029).

### 3.3. Results of Linear Mixed-Effects Model (Motor and Bulbar ALSFRS-R Scores)

The results of the linear mixed-effects models with motor or bulbar ALSFRS-R scores as dependent variables are shown in Table 4.

Similar to the total ALSFRS-R model, longer DDs were associated with lower initial motor and bulbar ALSFRS-R scores. As expected, the site of onset had a significant effect on the initial motor and bulbar ALSFRS-R scores.

In both the bulbar and motor ALSFRS-R models, changes in DD were correlated with changes in the ALSFRS-R slope. Each additional month of DD was associated with a slower decline of 0.013 points/month in the motor ALSFRS-R slope (*p* < 0.001) and a slower decline of 0.0045 points/month in the bulbar ALSFRS-R slope (*p* = 0.005).

While older age at onset was associated with steeper bulbar ALSFRS-R slopes, with each additional year at onset corresponding with faster decline by 0.0027 points/month (*p* = 0.008), it did not significantly affect the motor ALSFRS-R slope.

The site of onset did not significantly affect the bulbar ALSFRS-R slope. However, while no differences were found in the rate of motor ALSFRS-R slope between BO and ULO PALS, LLO PALS had a significantly slower rate of motor ALSFRS-R decline when compared to BO patients (difference of 0.236 points/month, *p* < 0.001).

When the models were re-run with only the ULO and LLO groups, motor ALSFRS-R declined more slowly in the LLO group (difference of 0.128 points/month, *p* = 0.017). There was no difference in the rate of decline of the bulbar ALSFRS-R score between those two groups (*p* = 0.498).

## 4. Discussion

ALS is a highly heterogeneous disease, and identifying progression patterns in clinically distinct subgroups may aid in prognostication, patient stratification in trials, and treatment selection. In this study, we used data collected in an ALS clinic in a tertiary medical center and combined several previous approaches to modeling ALS progression.

We found that patients with different sites of onset (BO, ULO or LLO) exhibit differences in DD and the rate of functional decline. Specifically, PALS with LLO had longer DDs than PALS with BO. Additionally, PALS with LLO tended to have slower declines in the total and motor ALSFRS-R scores compared to PALS with ULO and PALS with BO. These findings suggest that heterogeneity exists within the SO group and that analyzing PALS with ULO and LLO separately may be useful in stratifying PALS in clinical trials.

Our observation that PALS with BO had shorter DDs than PALS with LLO is consistent with previous reports comparing BO to SO as a whole [15,16,17]. However, we found no significant difference between the DD of the ULO and BO groups. Thus, the differences in DD between SO and BO groups may reflect differences in DD between the BO group and LLO group, rather than the SO group as a whole; this suggests a benefit of subdividing the SO group into ULO and LLO. One possible explanation for longer DD in the LLO group is that bulbar symptoms or dominant-hand weakness may be more debilitating than lower limb weakness, leading to an earlier referral to a specialist.

Regarding the rate of functional decline: although it has been reported that PALS with SO have slower progression rates than patients with BO [10,18], few studies performed an analysis that separated the ULO and LLO subgroups. Notably, a French study including 1844 PALS found that ALSFRS-R declined more rapidly in LLO patients (magnitude of difference not stated) [18], which contrasts with our findings, in which ULO patients had a faster decline.

Previous studies showed that ALS progression is primarily contiguous, especially in PALS with SO (93.9%) [19]. Moreover, the next affected site in PALS with LLO is likely to be the upper limbs, while in PALS with ULO, the next presenting site is likely to be the lower limbs [14]. It has also been shown that in both UMN- and LMN-predominant cases, ALS was more likely to progress caudally than rostrally (*p* < 0.0025). In addition, in PALS with UMN onset, the rate of progression from upper limbs to lower limbs was faster than the reverse (*p* < 0.0025) [14]. Therefore, the faster functional decline in PALS with ULO, which was mainly caused by the decline in the motor domain according to our data, may be partly explained by more significant lower limb involvement in this population (compared to upper limb involvement in the LLO group).

Our study population is similar to previously published cohorts—the overall male-to-female ratio in our study was 1.78, and the mean age was 57.7 [10,15,20].

In the BO group, the mean age was significantly higher than in the other groups, in line with previous publications [9,21]. Age was also a significant variable, affecting the total ALSFRS-R slope, and higher age at onset was associated with greater rate of decline (*p* = 0.005), which is also consistent with previous data [9,10,18]. In the linear models for the motor and bulbar subscales, higher age at onset was associated with a faster decline in the bulbar subscale (*p* = 0.008), but not in the motor subscale, suggesting that the effect of age is more significant in the bulbar component of the ALSFRS-R score. These findings demonstrate that age should be considered as an additional confounding variable when comparing PALS with different sites of onset.

According to the results of the linear mixed-effects model for the total ALSFRS-R (Table 3), longer DDs were significantly associated with lower total ALSFRS-R scores at the initial visit, and were also associated with less steep slopes. This supports previous reports that suggest that the functional decline in ALS is more rapid at the beginning of the disease, with a decrease in the slope in later stages [10,18]. Similar trends were observed in the linear models for the bulbar and motor subscales (Table 4), suggesting that longer DD is connected with greater functional deterioration at presentation in both of those domains.

Previous studies have identified additional variables that may be related to the pathogenesis of ALS and the prognosis of PALS. For example, air pollution has been shown to be associated with increased prevalence of ALS [22]. In addition, occupational history has been shown to be associated both with survival and with the site of onset in PALS [23]. Finally, weight loss at diagnosis has been associated with faster ALSFRS-R decline [24]. Future studies are required to evaluate the interplay of those variables in the pathogenesis and the progression of ALS. Previous studies have also identified the mTOR pathway [25] as a possible contributor to the progression of ALS. mTOR is a central regulator of cellular growth, metabolism, protein synthesis, and autophagy [26,27,28,29,30]. In the context of ALS, the ability of mTOR to inhibit autophagy may prevent the elimination of misfolded proteins, thus increasing the burden of the disease [25].

This study has several limitations. Firstly, it should be noted that ALSFRS-R has been shown to decline in an approximately linear fashion, and several previous studies have used linear mixed-effects models to assess the ALSFRS-R slope [8,10,31,32]. However, according to previous data, ALSFRS-R may decline in a curvilinear fashion, so that a faster rate of decline is observed at the beginning of the disease, especially in the first 18 months, and toward the end stages of the disease [18]. In light of this, we did not try to model the disease progression in the early stages of the disease. Instead, the model included ALSFRS-R measurements from the first visit to an ALS clinic in a tertiary medical center (the time from onset to initial visit was 17.2 months on average) with a maximum cutoff of 5 years since disease onset. Secondly, due to the retrospective nature of this study, recall bias may affect the accuracy of disease onset.

In conclusion, our data suggests that analyzing PALS with ULO and LLO separately may be useful in the stratification of PALS. For example, different results were obtained with regard to DDs when each of those groups was compared to PALS with BO, a finding that was not previously reported to the best of our knowledge. In addition, a slower motor and total function decline was noted in the LLO group in comparison with the ULO and BO groups. This observation might prove useful for future stratification of patients in clinical trials, as well as for identifying additional factors to consider when building machine learning-based models for the classification of PALS.

## Figures and Tables

**Table 1:** Patient characteristics at the initial visit.

	Upper Limb Onset (*n* = 100)	Lower Limb Onset (*n* = 108)	Bulbar Onset (*n* = 73)	*p* Value
Sex (males), *n* (%)	73 (73.0%)	63 (57.4%)	44 (60.3%)	0.058
Age at disease onset (years; mean ± SD)	55.7 (±12.7)	56.4 (±11.4)	62.2 (±11.7)	0.002
Diagnostic delay (months; mean ± SD)	11.1 (±8.1)	12.0 (±8.4)	8.7 (±6.4)	0.031 *
Total ALSFRS-R score at initial visit (mean ± SD)	37.0 (±7.8)	35.9 (±7.5)	35.9 (±7.2)	0.195
Motor ALSFRS-R at initial visit (mean ± SD)	15.1 (±5.7)	13.7 (±5.4)	18.7 (±4.8)	<0.001 *
Bulbar ALSFRS-R at initial visit (mean ± SD)	10.8 (±1.9)	10.6 (±2.0)	6.4 (±2.8)	<0.001 *
Respiratory ALSFRS-R at initial visit (mean ± SD)	11.4 (±1.7)	11.3 (±1.7)	10.8 (±1.7)	0.044 *
Treatment with Riluzole, *n* (%)	89 (89.0%)	88 (81.5%)	59 (80.8%)	0.235
Motor neuron involvement at site of onset, *n* (%)				<0.001 *
UMN	1 (1.0%)	5 (4.8%)	6 (8.3%)	
LMN	31 (32.0%)	17 (16.3%)	1 (1.4%)	
Mixed	65 (67.0%)	82 (78.8%)	65 (90.3%)	
Unknown	3	4	1	

One-way ANOVA tests were performed for continuous variables. Fisher’s exact test was performed for sex and motor neuron involvement at site of onset. ALSFRS-R—revised amyotrophic lateral sclerosis functional rating scale; SD—standard deviation. * *p* < 0.05.

**Table 2:** Results of Tukey’s HSD post hoc tests for comparison of patient characteristics at the initial visit.

Variable	Comparison Group 1 (Site of Onset)	Comparison Group 2 (Site of Onset)	Group 1 Value	Group 2 Value	Difference	Adjusted *p* Value
Diagnostic delay (months)	Upper limb	Lower limb	11.12	12.04	−0.92	0.673
	Upper limb	Bulbar	11.12	8.70	2.42	0.111
	Lower limb	Bulbar	12.04	8.70	3.34	0.014 *
Age at disease onset (years)	Upper limb	Lower limb	55.68	56.42	−0.74	0.896
	Upper limb	Bulbar	55.68	62.21	−6.53	0.001 *
	Lower limb	Bulbar	56.42	62.21	−5.79	0.004 *
Motor ALSFRS-R at initial visit	Upper limb	Lower limb	15.09	13.69	1.40	0.144
	Upper limb	Bulbar	15.09	18.71	−3.62	<0.001 *
	Lower limb	Bulbar	13.69	18.71	−5.02	<0.001 *
Bulbar ALSFRS-R at initial visit	Upper limb	Lower limb	10.81	10.63	0.18	0.825
	Upper limb	Bulbar	10.81	6.36	4.45	<0.001 *
	Lower limb	Bulbar	10.63	6.36	4.27	<0.001 *
Respiratory ALSFRS-R at initial visit	Upper limb	Lower limb	11.43	11.25	0.18	0.723
	Upper limb	Bulbar	11.43	10.75	0.68	0.026 *
	Lower limb	Bulbar	11.25	10.75	0.50	0.130

ALSFRS-R—revised amyotrophic lateral sclerosis functional rating scale.* *p* < 0.05.

**Table 3:** Results of linear mixed-effects model (total ALSFRS-R score).

	Coefficient Estimates	95% CI	Sattwerwaite *p* Value
Individual covariates ^			
Time since initial visit (months)	−1.16	[−1.33, −0.99]	NA
Age at disease onset (years)	−0.01	[−0.09, 0.07]	0.775
Diagnostic delay (months)	−0.19	[−0.30, −0.07]	0.002 *
Upper limb onset ^&^	2.32	[−0.10, 4.75]	0.061
Lower limb onset ^&^	0.42	[−1.97, 2.82]	0.729
Interaction term ^#^			
Upper limb onset ^&^	0.043	[−0.179, 0.266]	0.701
Lower limb onset ^&^	0.244	[0.030, 0.461]	0.027 *
Age at disease onset (years)	−0.099	[−0.017, −0.003]	0.005 *
Diagnostic delay (months)	0.020	[0.009, 0.031]	<0.001 *

^ Represents effects on the total ALSFRS-R score at the initial visit (intercept of model). Positive values indicate higher total ALSFRS-R at the initial visit. ^#^ Represents effects on the total ALSFRS-R slope (slope of model). The values denote changes in the total ALSFRS-R decline per month since the initial visit. Positive values indicate slower rates of decline. ^&^ Compared to people with ALS with bulbar onset. ALSFRS-R, revised amyotrophic lateral sclerosis functional rating scale; CI, confidence interval. * *p* < 0.05.

**Table 4:** Results of linear mixed-effects model (motor and bulbar ALSFRS-R subscores).

	Motor ALSFRS-R Score Coefficient Estimate	Motor ALSFRS-R Score Satterwaite *p* Value	Bulbar ALSFRS-R Score Coefficient Estimate	Bulbar ALSFRS-R Score Satterwaite *p* Value
Individual covariates ^				
Time since initial visit (months)	−0.74	NA	−0.24	NA
Age at disease onset (years)	0.007	0.808	−0.015	0.179
Diagnostic delay (months)	−0.103	0.017 *	−0.050	0.004 *
Upper limb onset ^&^	−3.41	<0.001 *	4.87	<0.001 *
Lower limb onset ^&^	−4.85	<0.001 *	4.69	<0.001 *
Interaction term ^#^				
Upper limb onset ^&^	0.107	0.119	0.005	0.883
Lower limb onset ^&^	0.236	<0.001 *	0.020	0.542
Age at disease onset (years)	−0.0037	0.084	−0.0027	0.008 *
Diagnostic delay (months)	0.013	<0.001 *	0.0045	0.005 *

^ Represents effects on bulbar/motor ALSFRS-R score at the initial visit (intercept of model). Positive values indicate higher bulbar/motor ALSFRS-R at the initial visit. ^#^ Represents effects on bulbar/motor ALSFRS-R slope (slope of model). The values denote changes in bulbar/motor ALSFRS-R decline per month since the initial visit. Positive values indicate slower rates of decline. ^&^ Compared to people with ALS with bulbar onset. ALSFRS-R, revised amyotrophic lateral sclerosis functional rating scale. * *p* < 0.05.

## Data Availability

The data that support the findings of this study are available on request from the corresponding author. The data are not publicly available due to privacy or ethical restrictions.

## Supplementary Materials

The following supporting information can be downloaded at: https://www.mdpi.com/article/10.3390/jcm15083096/s1, Supplementary Table S1: AIC (Akaike information criterion) metric for different tested linear mixed effects models.
