# Supplementary material for: Slower Progression Rates in Lower Limb-Onset ALS
Source: J Clin Med. 2026 Apr 18;15(8):3096. doi: 10.3390/jcm15083096 (PMC13116631; doi:10.3390/jcm15083096)
Supplement: Supplementary file 1 [file jcm-15-03096-s001.zip › jcm-4205107-supplementary.pdf]

Supplementary Table S1. AIC (Akaike information criterion) metric for different tested linear mixed effects models.

**Model**	**AIC**
Time since initial visit + diagnostic delay + onset site	**7593.2**
Time since initial visit + diagnostic delay + onset site + sex	**7594.8**
Time since initial visit + diagnostic delay + onset site + age	**7589.1**
Time since initial visit + diagnostic delay + onset site + age + sex	**7590.9**
